# Integrated Expression Profiling and ChIP-seq Analyses of the Growth Inhibition Response Program of the Androgen Receptor

**DOI:** 10.1371/journal.pone.0006589

**Published:** 2009-08-11

**Authors:** Biaoyang Lin, Jun Wang, Xu Hong, Xiaowei Yan, Daehee Hwang, Ji-Hoon Cho, Danielle Yi, Angelita G. Utleg, Xuefeng Fang, Dustin E. Schones, Keji Zhao, Gilbert S. Omenn, Leroy Hood

**Affiliations:** 1 Department of Urology, University of Washington, Seattle, Washington, United States of America; 2 Zhejiang-California International NanoSystems Institute, Zhejiang University, Hangzhou, Zhejiang, China; 3 Swedish Medical Center, Seattle, Washington, United States of America; 4 The Institute for Systems Biology, Seattle, Washington, United States of America; 5 I-Bio Program & Department of Chemical Engineering, Pohang University of Science and Technology, Pohang, Kyungbuk, Republic of Korea; 6 Laboratory of Molecular Immunology, National Heart, Lung, and Blood Institute, National Institutes of Health, Bethesda, Maryland, United States of America; 7 Center for Computational Medicine and Biology, University of Michigan, Ann Arbor, Michigan, United States of America; Baylor College of Medicine, United States of America

## Abstract

**Background:**

The androgen receptor (AR) plays important roles in the development of male phenotype and in different human diseases including prostate cancers. The AR can act either as a promoter or a tumor suppressor depending on cell types. The AR proliferative response program has been well studied, but its prohibitive response program has not yet been thoroughly studied.

**Methodology/Principal Findings:**

Previous studies found that PC3 cells expressing the wild-type AR inhibit growth and suppress invasion. We applied expression profiling to identify the response program of PC3 cells expressing the AR (PC3-AR) under different growth conditions (i.e. with or without androgens and at different concentration of androgens) and then applied the newly developed ChIP-seq technology to identify the AR binding regions in the PC3 cancer genome. A surprising finding was that the comparison of MOCK-transfected PC3 cells with AR-transfected cells identified 3,452 differentially expressed genes (two fold cutoff) even without the addition of androgens (i.e. in ethanol control), suggesting that a ligand independent activation or extremely low-level androgen activation of the AR. ChIP-Seq analysis revealed 6,629 AR binding regions in the cancer genome of PC3 cells with an FDR (false discovery rate) cut off of 0.05. About 22.4% (638 of 2,849) can be mapped to within 2 kb of the transcription start site (TSS). Three novel AR binding motifs were identified in the AR binding regions of PC3-AR cells, and two of them share a core consensus sequence CGAGCTCTTC, which together mapped to 27.3% of AR binding regions (1,808/6,629). In contrast, only about 2.9% (190/6,629) of AR binding sites contains the canonical AR matrix M00481, M00447 and M00962 (from the Transfac database), which is derived mostly from AR proliferative responsive genes in androgen dependent cells. In addition, we identified four top ranking co-occupancy transcription factors in the AR binding regions, which include TEF1 (Transcriptional enhancer factor), GATA (GATA transcription factors), OCT (octamer transcription factors) and PU1 (PU.1 transcription factor).

**Conclusions/Significance:**

Our data provide a valuable data set in understanding the molecular basis for growth inhibition response program of the AR in prostate cancer cells, which can be exploited for developing novel prostate cancer therapeutic strategies.

## Introduction

Androgens and the androgen receptor (AR) play important biological roles in the development of male phenotype and urogenital tissues, including prostate, and in the initiation and progression of many human diseases. Alterations in AR sequences and expression levels and perturbations in AR signaling networks have significant roles in the genesis and maintenance of prostate cancers (PCa) [1]–[6]. Prostate cancers are often treated with hormone deprivation strategies such as orchidectomy, a luteinizing hormone-releasing hormone (LHRH) analogue, or an antiandrogen. Although they are initially responsive to the treatment strategy, nearly all prostate cancers progress to ‘hormone-refractory’ or ‘androgen-independent’ disease. There are data suggesting that the AR signaling is still functional in ‘hormone-refractory’ prostate cancer (HRPC). The mechanisms include 1) activation of the AR by other growth factors [7]; 2) mutations in the AR that change AR ligand specificity and allow AR to be activated by non-steroids or anti-androgens [8]; 3) over-expression of the AR [2], [9]–[11]; 4) recruitment of new AR-cofactors [12]. These data suggest that the AR seems to act as a promoter of carcinogenesis in HRPC.

In contrast, Yuan et al. showed an androgen-induced inhibition of cell proliferation in an androgen-insensitive prostate cancer cell line (PC3) transfected with a human androgen receptor complementary DNA. They showed that in PC3 cells expressing the transfected androgen receptor (PC3-AR) under the simian virus 40 (SV40) promoter, androgen decreased the proliferation rate and cloning efficiency and induced a more differentiated phenotype. Their data demonstrated that PC3 cells have retained the cellular machinery required to respond to the activated androgen receptor. Similar results were obtained showing that androgens suppress the proliferation of PC3-AR cells using two other promoters: cytomegalovirus (CMV) promoter [13] and the EF1a promoter [14].

Niu et al. recently found that AR is a tumor suppressor or a proliferator in prostate cancer depending on cellular contexts [15], [16]. Niu et al. studied the effects of AR in the PC3-AR9 cells, which are PC3 cells expressing the AR under the control of its own native promoter [17]. They found that restoring the AR in PC3 cells could suppress their *in vitro* and *in vivo* invasion capabilities. All these data generated in PC3 cells seem to contradict the traditional belief that the AR functions as a stimulator in prostate tumor growth and metastasis. As PC3 cells expressed the basal marker-CK5 [18], PC3 cells might have some basal cell properties, which make them different from other prostate cancer cell lines such as LNCaP, which expresses CK8/18 and shows luminal cell properties [18].

We hypothesize that PC3 cells possess cellular machinery that turn the AR in PC3-AR cells into growth suppressor. We applied expression profiling to identify the response program of PC3-AR cells to different growth conditions (i.e. with or without androgens and different concentration of androgens) and then applied the newly developed ChIP-seq technology to identify the AR binding regions under androgen deprivation conditions. Out data provide a valuable data set in understanding the molecular basis for the growth prohibitive response program of the AR in advanced prostate cancers. The growth prohibitive properties of the AR or its response program can be exploited for developing novel prostate cancer therapeutic strategies.

## Results

### PC3 cells expressing AR initiate ligand independent activation of the AR response program

In an effort to characterize androgen-induced growth inhibition mechanism and to identify targets that could be used to induce growth inhibition and differentiation in advanced prostate cancer cells, we used PC3 cells transfected with the wild-type AR as a model and performed gene expression profiling analysis. We compared PC3 cells with or without harboring the wild-AR construct in the growth conditions of 1 nM R1881, 10 nM R1881 and ethanol (the solvent for R1881). The MOCK control is PC3 cells transfected with empty vectors.

**Figure 1:**
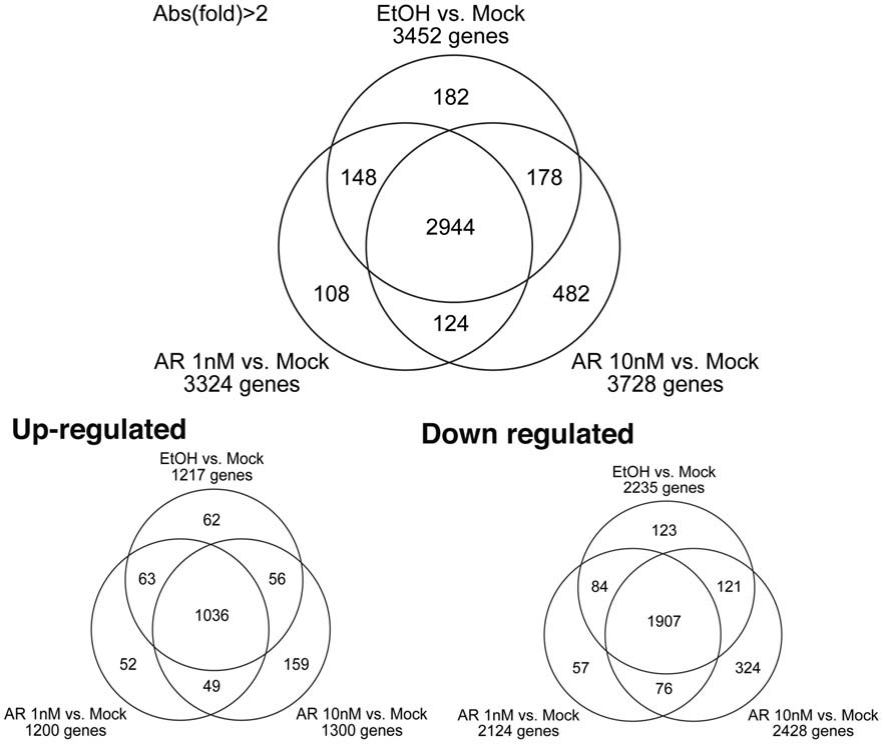
Venn diagrams showing the number of genes regulated in the three comparisons (ETOH vs. Mock, 1 nM vs. Mock, 10 nM vs. Mock) (the Top Panel), and the number of up or down regulated genes in each comparison (the Bottom Panel). We noted that two genes (S100A4 and MME) are up regulated in the condition of 1 nM R1881 but down regulated in the ethanol condition comparing to the MOCK control, and one gene HSPD1 is up-regulated in the condition of 10 nM R1881 but down regulated in ethanol. It causes the disagreement of number of genes between Venn-diagrams in the top panel and that of the lower panel.

To our surprise, the comparison of MOCK-transfected PC3 cells with AR-transfected cells showed 3,452 genes with differential expression (two fold cutoff) even without addition of androgens (i.e. in ethanol control) (Table S1 and Figure 1). A carefully repeated biological replicate showed consistent results. This suggests that other factors than androgens could initiate AR-mediate response without the canonical AR binding ligand androgens. Alternatively, as we used charcoal-stripped serum, which still has about 10% of androgens remaining in the serum [7], it may suggest that low levels of androgen can still activate a large number of genes through AR in the advanced prostate cancer cell PC3. Among these 3,452 genes, 2235 are down regulated genes and 1217 up regulated genes in AR-PC3 cells compared to MOCK-transfected cells (Table S1). Adding 1 nM androgen only adds 232 differentially expressed genes (two fold cutoff, 133 down regulated and 101 up regulated). Adding 10 nM R1881 (androgens) generated an additional 482 differentially expressed genes (324 down regulated and 159 up regulated) (Figure 1). The union of all three comparisons generated a total list of 4,166 genes that can be regulated by androgens in various concentrations (Table S1). The array data were submitted to Gene Expression Omnibus (GEO) with accession number GSE15091 (http://www.ncbi.nlm.nih.gov/geo/query/acc.cgi?acc=GSE15091).

### Functional characterization of the differentially expressed genes between PC3-AR cells and Mock-transfected cells

Using GOminer analysis [19], we found that, in the up-regulated genes in PC3-AR cells compared to Mock-transfected cells, the most significantly enriched GO term is negative regulation of biological process (GO:0048519), suggesting a general down-regulation of biological process in AR-PC3 cells, which is consistent with the observation that over-expression of AR in PC3 cells inhibits cell proliferation [20]. Other interesting enriched GO terms include GO:0008283 cell proliferation, GO:0012501 programmed cell death, GO:0007249 I-kappaB kinase NF-kappaB cascade, GO:0007259 JAK-STAT cascade, GO:0042509 regulation of tyrosine phosphorylation of STAT protein (Table 1 and table S2).

**Table 1:** Enriched GO terms in up regulated list in PC3-AR cells compared to MOCK-transfected cells.

GO CATEGORY	Total Genes	Changed Genes	Enrichmentt	LOG10(p)
GO:0008283_cell_proliferation	507	58	1.47	−2.86
GO:0012501_programmed_cell_death	425	67	2.03	−8.15
GO:0006915_apoptosis	423	67	2.04	−8.24
GO:0007249_I-kappaB_kinase_NF-kappaB_cascade	104	20	2.48	−3.94
GO:0045595_regulation_of_cell_differentiation	64	12	2.41	−2.48
GO:0007259_JAK-STAT_cascade	31	8	3.32	−2.70
GO:0042509_regulation_of_tyrosine_phosphorylation_of_STAT_protein	10	4	5.15	−2.29
GO:0042771_DNA_damage_response__signal_transduction_by_p53_class_mediator_resulting_in_induction_of_apoptosis	5	3	7.73	−2.38

The GO terms enriched by the down regulated genes include GO terms involved in transport and cellular localizations, and in general metabolic process such as the TCA (GO:0006099 tricarboxylic acid cycle) cycle, which again is consistent with the growth inhibition phenotype observed (Table 2 and table S2).

**Table 2:** Enriched GO terms in down regulated list in PC3-AR cells compared to MOCK-transfected cells.

GO CATEGORY	Total Genes	Changed Genes	Enrichmentt	LOG10(p)
GO:0046907_intracellular_transport	282	53	1.56	−3.32
GO:0006892_post-Golgi_vesicle-mediated_transport	14	7	4.16	−3.25
GO:0006099_tricarboxylic_acid_cycle	7	4	4.76	−2.27
GO:0046356_acetyl-CoA_catabolic_process	7	4	4.76	−2.27
GO:0009081_branched_chain_family_amino_acid_metabolic_process	6	5	6.94	−3.87

### Identification of global ligand-independent AR binding sites by ChIP-seq analysis in PC3-AR cells

In order to identify the direct targeted genes of AR in the ligand-independent AR response program, we performed ChIP-seq of the DNAs from anti-AR chromatin IP, as described by Barski et al. [21]. Through commercialization of the second generation of high throughput technologies (e.g. from Illumina-Solexa, Roche and ABI/SOLiD), ChIP-seq technology is rapidly emerging as a cost effective, global technology for high-resolution, genome-wide, unbiased mapping of protein-DNA interactions in chromatin-mapped complexes. It has been applied in global identification of in vivo binding of the insulator binding protein, CTCF [21], the neuron-restrictive silencer factor (NRSF) and STAT1 [22], [23]. Ji et al. recently showed that ChIP-seq has better ability than ChIP-Chip in identifying TF binding regions for NRSF [24]. Therefore, we adopted the ChIP-seq approach in this study.

The same transfected PC3 cells as we used for gene expression profiling were used for ChIP-seq analysis. Transfected PC3 cells were grown without the addition of androgens. After ChIP-seq analysis, we obtained a total of 5,354,469 20-nucleotide (nt) sequence tags that can be mapped uniquely to the human genome allowing up to two mismatches to accommodate normal polymorphisms. We also performed ChIP-seq analysis for IgG negative controls and obtained 1,089,089 tags.

**Figure 2:**
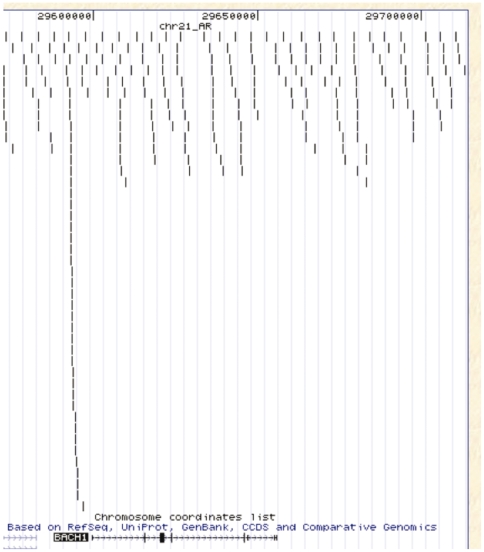
An example of the mapping of the ChIP-seq tags to a region in the human chromosome 21. Each black bar represents a unique ChIP seq taq mapped to the region. The promoter region of the BACH1 (BTB and CNC homology 1, basic leucine zipper transcription factor 1) contains a peak of ChIP-seq tag alignments to this chromosomal region, suggesting that the AR binds to this promoter. The figure was generated by uploading to the UCSC genome browser a file containing all unique ChIP seq tags in the BED format. Annotations are from the UCSC genome browser.

Figure 2 shows an example of the mapping of ChIP-seq data on the promoter region of the gene *BACH1* on chromosome 21. We used the CisGenome program [24] to systematically identify DNA binding regions of AR. Analysis using the EXPLORE algorithm of the CisGenome showed a strong enrichment for AR ChIP reads (with the dP0 hat at 0.7526). Using an FDR (false discovery rate) cut off of 0.05, we identified a total of 6,629 AR binding regions in the PC3 cells (Table S3). Among them, 4435 AR binding regions (about 67%) can be mapped to 3592 genes with unique Refseq IDs using the closest distances of 50 kb to the TSS start or end sites as criteria, suggesting that a gene can contain multiple AR binding regions. Using Entrez IDs as identifiers, 4435 AR binding regions (about 67%) can be mapped to 3316 unique genes with Entrez IDs. The average number of AR binding regions per gene is 1.26. The rest of them (2,194 AR binding regions, about 33%) could be mapped to more than 50 kb away from the transcript starts or ends of known genes or to regions that have not yet been annotated to be associated with genes in the human genome.

The top 10 mostly strong AR binding regions by the number of sequence tags mapped contain the genes for ATIC (IMP cyclohydrolase), TUSC1 (Tumor suppressor candidate 1), AQP2 (Aquaporin 2), GFRA1 (GDNF family receptor alpha 1), PACRG (PARK2 co-regulated), OR1J1 (Olfactory receptor, family 1, subfamily J, member 1) and genes with unknown functions such as KIAA0350 and FAM118A (Family with sequence similarity 118, member A). Interestingly, among the top ten AR binding regions, there are two AR binding regions (chr2: 19587584–19587634 and chr16: 74498964–74498992) that seem not mapped to any known genes. However, chr16: 74498964–74498992 is about 46 kb away form spliced EST BX094831, and chr2: 19587584–19587634 is about 35 kb from spliced EST BG219799, suggesting that these two genes may be the genes yet to be fully characterized and possibly regulated by the AR.

To confirm that ChIP-seq is able to identify AR binding in genes, we randomly picked 5 genes and confirmed enrichment of 4 genes in the ChIP DNA pulled down by the AR antibody compared to ChIP DNA pulled down by the mouse IgG control (Table 3). The FDR rate calculated by this strategy is 20%.

**Table 3:** RT-PCR confirmation of AR binding regions in PC3-AR cells.

Gene Symbol	Description	RT-PCR Ratio AR ChIP/IgG ChIP
GSK3B	Glycogen synthase kinase 3 beta	3.03
OR9Q1	Olfactory receptor, family 9, subfamily Q, member 1	25.03
TADA2L	Transcriptional adaptor 2 (ADA2 homolog, yeast)-like	3.26
ZNF533	Zinc finger protein 533	3.26
ARL16	ADP-ribosylation factor-like 16	0.79

### Categorization of the AR binding regions identified by ChIP-seq based on their relative positions to genes

**Figure 3:**
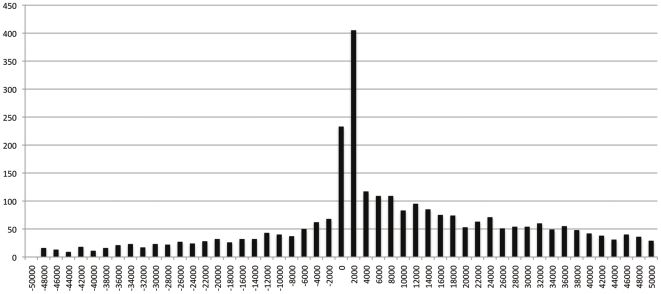
Histograms of AR binding sites around annotated TSS (Transcription start sites) (+ or −50 kb). Frequencies of AR island binding were calculated every two kilobases (Y-axis). Relative distance to TSS is shown in X-axis, Negative and positive values indicate localization 5′ or 3′ to TSS respectively.

We then mapped these binding regions to the human genome and categorized these binding regions based on their locations relative to the genes in the human genome using the CisGenome's annotation functions. The mapping was done with the gene annotations from the UCSC genome annotation database (09/05/2008) for the human genome (hg18, Build 36.1). We tabulated the frequencies of AR binding sites to the transcription start sites (TSSs) for every two kilo bases. A histogram of AR binding sites residing within the downstream 50 kb or upstream 50 kb genomic regions relative to annotated TSSs is shown in Figure 3. Most of the AR binding regions was identified around the TSS sites (Figure 3). About 22.4% (638 of 2,849) can be mapped to within 2 kb of the transcription start site (TSS).

### Correlation of AR binding with gene expression data

To answer the question whether ligand-independent AR binding to the genomic regions exerts functional consequences through changes in the expression level of the targeted genes, we integrated the ChIP-seq data with gene expression profiling data.

2163 AR binding genes (2679 AR binding regions) identified by ChIP-seq have corresponding genes on the array we used for expression profiling analysis. Comparing the expression profiling data with the ChIP-seq data, we identified a total of 727 genes (937 AR binding regions) for which the genes were changed by two fold in the ETOH vs. Mock comparison; among them, 543 genes (716 AR binding regions) were down regulated, and 184 genes (221AR binding regions) were up-regulated. Addition of 1 nM R1881 generated an additional 43 genes (28 down regulated and 15 up regulated), and addition of 10 nM R1881 identified a further 89 genes (66 down regulated and 23 up regulated). The union of the three analyses identified a total of 859 genes (1114 AR binding regions) (51.5%, 1,114/2,163,) that are differentially expressed at various concentrations of androgens by at least two fold (Table S3 and Figure 1, lower panel). Among the 859 genes that contain AR binding sites and are changed in expression levels in response to androgen treatments, 637 genes are down regulated while 222 genes are up regulated. We found that the androgen regulated genes in PC3-AR9 cells are significantly enriched for AR binding sites (with one-sided Fisher's exact test, P = 1.82e-11).

### Identification of ARE consensus in the AR binding regions

The androgen receptor regulates the transcription of specific target genes by binding to specific DNA response elements in their promoters, referred to as androgen response elements (AREs) [25], [26]. In the Transfac database, there are three AREs derived from *in vitro* binding data or from a limited numbers of human promoters: the M00481 (consensus: GGTACANNRTGTTCT)[25]; M00447 (consensus sequence AGWACATNWTGTTCT)[26]; M00962 (consensus sequence WGAGCANRN). To see how many AR motifs were found in the AR binding regions, we loaded the AR matrix (obtained from the Transfac TF database) tables for M00481, M00447 and M00962 into the CisGenome program to compare to all AR binding regions that we identified in PC3 cells with a likelihood (LR) ratio of 500 compared to genomic background. We identified 40, 21, 147 respectively for M00481, M00447 and M00962 (Table S4). The union of the above three gives 190 sites (about 7.1%, 190 out of 2,679 AR binding regions). Searching against the AR binding regions with the AR half-site motif TGTTCT with the criterion of allowing no mismatches, we identified 226 binding regions (about 8.4%) that contain the AR half-site motif.

**Figure 4:**
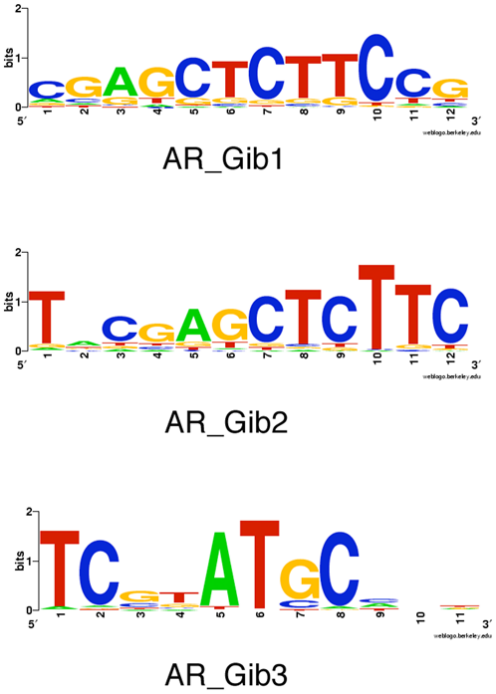
The consensus sequences of the three novel AR matrice identified, which can be mapped to 1,448, 1,012, 317 AR binding regions respectively. The consensus logos were generated with the Web logo program (http://weblogo.berkeley.edu/) using the mapped sequences from Table S5–S7.

Since the percentage of the ARE identified in our AR binding regions is low, we asked the question whether there exists novel AR binding motifs in the AR binding regions of PC3 cells. We hypothesize that the AR binding motif in the prohibitive responsive PC3-AR cells might be different from that in the proliferative prostate cancer cells. We therefore used the Gibbs Motif Sampler of the CisGenome program to identify novel AR binding motifs. With a likelihood (LR) ratio of 500 compared to AR IGG ChIP-Seq background, we identified five novel putative AR binding motifs. Two of these motifs are general GC rich or AT rich sequence motifs and may not be specific and were discarded. The reminding three new motifs were named AR new motif Gib1 to 3. AR new motif Gib1 to 3 can be mapped to 1448, 1012, 317 AR binding regions respectively (Table S5, S6, S7). The union of the AR binding regions containing one of these two motifs is 1,808 AR binding regions, i.e. 27.3% of AR binding regions, (1,808/6,629), which is much higher than the number of mapped AR binding regions by the known canonical AR motif from the Transfac database. The consensus logos of the new AR motifs are shown in Figure 4. They have a core sequence motif CGAGCTCTTC. We were curious whether the sequence motif shows any similarities to known binding motifs. Using JASPAR: an open-access database for eukaryotic transcription factor binding profiles (http://jaspar.genereg.net/)[27], we found that the highest ranked match is to NR3C1 with score of 14.5926 (72.96 percentage score). NR3C1 is the nuclear receptor subfamily 3, group C, member 1 (glucocorticoid receptor) gene. Interestingly, both AR and the NR3C1 belong to same SCOP (Structural Classification of Proteins) family ‘Nuclear receptor ligand-binding domain’ (http://scop.mrc-lmb.cam.ac.uk/scop/data/scop.b.b.cdh.b.b.html). They are very similar in structure, with the RMSD (root mean square deviation) between two structures being 1.35 angstrom although the sequence similarity is only 47%. However, a search using the NR3C1 matrix using the CisGenome program with the same criteria we used previously only identified 53 matches to NR3C1 motif, suggesting that the NR3C1 motif, although is the highest ranking matches of known TFs, is different from the new motifs we identified. The new motif we identified with the core sequence CGAGCTCTTC is truly a novel motif not previously defined.

We were curious whether other known TFs could be bind to the AR binding regions that we identified and act as AR cooperators for regulation of gene expression. In order to systematically search for potential bindings of other transcription factors, we used the MotifScanner program (http://homes.esat.kuleuven.be/~thijs/download.html) and scanned all TF motifs (PWM databases) using the human transcription factor subset of the Transfac professional 7.0. The matrices with the top ranking matches include TF matrix TEF1_Q6 (Transcriptional enhancer factor), GATA_Q6 (GATA transcription factors), OCT_Q6 (octamer transcription factors) and PU1_Q6 (PU.1 transcription factor) with frequencies of 7.8%, 6.3%, 5.2%, 5.2%, respectively. The result of motif scanning is provided as table S8. Interestingly, the matrices with the top 10 ranking matches contain three GATA TF matrices: GATA_Q6, GATA1_04 and GATA3_01 (Table S8), suggesting that GATA family matrix might be the one of the highest co-occurrence TF in the AR binding regions of PC3-AR cells.

## Discussion

We showed that AR could be activated in low androgen or ligand independent manner in advanced prostate cancer cell line PC3 cells expressing the AR. the comparison of vector-transfected PC3 cells with AR-transfected PC3 cells showed that 3,449 genes are differentially expressed (two fold cutoff) even without addition of androgens (Figure 1 and table S1). This observation could be due to remaining low level of androgens in the medium, or due to androgen-independent activation of AR, or a combination of both. Page et al. recently showed that in patients with medical castration, although there is a 94% decrease in serum T, intraprostatic T and dihydrotestosterone levels remained 20–30% of control values, and prostate cell proliferation, apoptosis, and androgen-regulated protein expression were unaffected. The concentration of androgens remains in our culture condition using charcoal striped serum will probably similar to those or lower than those obtained by medical castration as charcoal stripped serum still has about 10% of androgens remaining in the serum [7]. Yuan et al. found that DHT concentration as low as 0.1 nM was able to inhibit cell proliferation of PC3 cells expressing AR by more than 40% after 72 h of incubation [20].

The PC3-AR expression model is in contrast to the androgen-sensitive and widely used LNCaP cell line, which, although expresses AR, but harbors a mutated AR (a missense mutation T877A) that confers promiscuity in its ligand binding. The mutated AR in LNCaP cells can be activated not only by androgens but also by progesterone, estrogen, adrenal androgens, and hydroxyf lutamide [28], [29]. The PC-3 was initiated from a bone metastasis of a grade IV prostatic adenocarcinoma from a 62-year-old male Caucasian [30]. Several studies demonstrated that PC PC3 cells expressing the transfected androgen receptor (PC3-AR) show growth inhibition and reduced invasion capabilities [13]–[16], [20]. Gene Ontology analysis of differentially expressed genes demonstrated that the activated AR program in PC3-AR cells are mostly involved in negative regulation of biological process, which is consistent with the growth inhibition properties of pC3-AR cells observed previously. Comparison of the AR regulated DEGs from our PC3-AR expression model with two AR GEGs published previously by Deprimo et al. [31] and Nelson et al. [32] from LNCaP cells revealed that the overlap is not significant (one-sided Fisher's exact test P = 0.9996, and the p-value from Gaussian fitting of 10,000 random sampling is 1), again suggesting that the AR response programs in these two model systems are different. We hypothesize that the different sets of AR co-regulators between LNCAP and PC3-AR cells can explain the difference in the AR response programs between the PC3-AR and the LNCaP models. Cell-line and tissue-specific androgen receptor-coregulators have been documented by Bebermeier et al. [33].

We applied ChIP-seq technology to build a genome-wide high-resolution map of the AR binding regions in PC-3 cell line transfected with the AR. About 22.4% (638 of 2849) of the AR binding regions in PC3-AR cells can be mapped to within 2 kb of the transcription start site (TSS) (Figure 2) and about 62.2% (2,849 of 4,435) of the AR binding regions could be mapped to within 50 kb of transcription start or end sites of annotated genes based on UCSC genome annotation (hg18). Bolton et al. designed tiling arrays around the transcription start sites of 548 candidate hormone-responsive genes and they identified 524 AR binding regions using ChIP to chip analysis [34]. They found that only 67% of the 524 AR binding regions they identified were within 50 kb of the TSS of the androgen regulated genes [34]. Transcriptional factor (TF) binding sites can be localized to regions other than the 5′ proximal promoter regions, such as far upstream sequences, introns, and 3′ of the gene as exemplified by genome-wide localization analysis (GWLA) of two transcription factors (p53 and estrogen receptor) [35], [36]. There is no consensus about how far a TF can bind upstream of a TSS and still be considered as its cis-regulatory TF of a given gene. Values ranging from 1 kb to 100 kb of TSS have been used by others [35], [36], reflecting limited understanding of transcription factor binding. The AR is known to be able to regulate genes from a far distance. For example, Magee et al. showed that androgens directly regulate FKBP5 via an interaction between the AR and a distal enhancer located 65 kb downstream of the transcription start site in the fifth intron of the FKBP5 gene [37]. Recently, Jariwala et al. discovered 19 novel loci occupied by the AR in castration-resistant C4-2B PCa cells by chromatin immunoprecipitation (ChIP) display. They found that only four of the 19 AR-occupied regions were within 10-kb 5′-flanking regulatory sequences, while three were up to 4-kb 3′ of the nearest gene, eight were intragenic, and four were in gene deserts [38]. Marioni et al. used RNA-seq to map deep sequencing of mRNAs to the human genome; they found that a significant portion of the mRNA sequences fall near an annotated gene but not in the annotated region [39], which suggests that annotations of many genes in the Ensembl or UCSC require extension or revision. This imprecision may affect mapping statistics of our AR binding data to the human genome. In addition, they showed that a sizeable fraction (10.6%) mapped to locations at least 100 kb from a known gene. This observation, together with the published data from the ENCODE Project Consortium [40], suggests that many transcriptionally active regions (TARs) are currently unannotated [39]. Some of our AR binding sites that we found to be localized in intergenic regions may be localized to these TARs. Over expression of AR in PC3 cells will not necessary result in non-specific binding in a ChIP as the specificity of binding in a ChIP experiment is determined by antibody specificity and by the washing conditions. We have optimized the ChIP conditions and identified an AR antibody that yielded high specificity in the ChIP experiments (Figure S1).

We also compared our data to a global AR binding data set from Massie et al., who identified 1,532 potential AR-binding sites in AR responsive and proliferative LNCaP cells using the NimbleGen promoter array that contains 1.5 kb promoter regions of about 25K human genes [41]. As Massie et al. used only 1.5 kb promoter, we compare 208 AR binding regions that localized within 1.5 kb of the 5′ of TSS sites, and we found that only 19 (9.1%) also mapped to the 1532 AR promoter regions in LNCaP cells (Table S9). A one-sided Fisher's exact test suggested that the overlap is not significant (P = 0.92). Wang *et al.* used ChIP to chip to identify and map 90 *AR* binding sites on chromosomes 21 and 22 in a prostate cancer cell line LNCaP under the treatment of 100 nM of androgen 5a-dihydrotestosterone (DHT) for 1 hour and 16 hours [12]. These 90 *AR* binding sites could be mapped to 27 genes; we identified only three of them (TMEM50B, BTG3 and MORC3) overlapping with our ChIP-seq data. The big difference between our ChIP-seq data and other's data is probably due to several factors. First, different technologies (ChIP-seq v.s. ChIP-chip) were used. Using NRSF as an example, Ji et al. showed that 5,517 (78%) of 7,114 ChIP-chip peaks did not overlap with the ChIP-seq peak [24]. They showed 933 (58.8%) of the 1,587 peaks common to ChIP-seq and ChIP-chip contain the NRSF motif, and 20.9% of peaks identified by ChIP-seq but not found by arrays contain the NRSF motif. However, only 68 (1.23%) of 5,517 ChIP chip specific peaks contain the NRSF motif [24]. Second, AR binding may require co-factors that are different in different cells. The cells used in these studies are different: Massie et al. used LNCaP cells [12], [41] while we used PC3 over-expressing the wild type AR. Finally, both lists may contain come false positives, which could reduce the chance of overlap. We used a transient expression of AR in PC3 cells, which might cause over representation of AR bindings.

We integrated the ChIP data with the gene expression data of PC3-AR cells in response to androgens. We found that 859 AR binding genes (1114 AR binding regions) (51.5%, 1,114/2,163,) are differentially expressed at various concentrations of androgens by at least two fold (Table S3 and Figure 1, lower panel). Other AR binding genes may also be regulated by androgens by less than two fold, of course. There is also a possibility that AR binding is a pre-condition for androgen response, and other factors may be needed for some promoters to turn AR binding into AR-response (i.e changed expression levels).

Some of these AR binding regions are adjacent to genes that are up regulated and others are adjacent to genes that are down regulated by androgen (Table S1). This is consistent with the result by Jariwala et al., who showed that, of 32 genes within 100 kb from the 19 novel AR-occupied regions they identified by ChIP, ten were stimulated by the AR but four were repressed in DHT-treated C4-2B cells [38]. It is also possible that, for some promoters, AR binding only poises the promoters for additional binding of other factors, just like what Jia et al. showed that AR-occupied regions in prostate cancer cells exceed number of DHT-responsive genes [42]. We noticed that AR did bind to the promoter regions of KLK3 (PSA) gene (Table S3) and confirmed its enrichment in the AR-ChIP compared to IgG-IP (Figure S1), but the expression level of KLK3 did not change even in the presence of 1 nM or 10 nM of R1881, suggesting that other co-factors are need to turn binding events into functional consequences of changed gene expressions for some genes like KLK3.

We identified the top ranking co-occupancy transcription factors for TEF1_Q6 (Transcriptional enhancer factor), GATA_Q6 (GATA transcription factors), OCT_Q6 (octamer transcription factors) and PU1_Q6 (PU.1 transcription factor). Wang et al. identified GATA2 and Oct1 as cooperators of AR in mediating the androgen response in LNCaP cells [12]. TEF1 and PU1 are two putative novel AR cooperators that we identified for PC3-AR cells. TEF1 is also named TEAD1 (TEA domain family member 1, SV40 transcriptional enhancer factor). Recently, Knight et al found that TEAD1 is novel prostate basal cell markers that correlate with poor clinical outcome in prostate cancer [43]. Knockdown TEAD1 expression using siRNA in prostate cell lines led to decreased cell growth in PC3 and disrupted acinar formation in a 3D culture system of RWPE1. However, the interaction of TEAD1 and AR has not been studied and future investigation is warranted. PU1 is also named hematopoietic transcription factor, and it is ETS-domain transcription factor that activates gene expression during myeloid and B-lymphoid cell development [44]. Its role in prostate cancer or its relationship with AR has not been studied.

We found that 190 sites (about 2.9%, 190 out of 6,629 AR binding sites) contain the proliferative AR consensus matrix M00481, M00447 and M00962 (from the Transfac database) with a likelihood (LR) ratio of 500 compared to genomic background. Our data is similar to Wang et al.'s findings that only 10% of the binding regions that they identified have a canonical class I NR (AGAACAnnnTGTTCT) binding motif when allowing up to two positions to vary from the palindromic consensus with 3 nucleotide spacing [12]. Massie et al. observed that 410 of 1,532 AR binding regions (26.8%) contain the ARE sequence motif M00481 (consensus: GGTACANNRTGTTCT) [41]. We have identified a lower percentage of AR binding regions contain the AR consensus site, probably due to the stringent criterion we used, or suggesting that the AR binding consensus for the prohibitive response in PC3-AR cells is different from that of the proliferative cells such as LNCaP cells. This maybe not surprising as the consensus sequences for the AR in the Transfac database are derived either from *in vitro* binding data or from a limited numbers of human promoters: the M00481 (consensus: GGTACANNRTGTTCT) was built from 29 binding sites selected from random oligonucleotides [25]; M00447 (consensus sequence AGWACATNWTGTTCT) was derived from AR binding sites in mouse *crisp3* (cysteine-rich secretory protein 3)[26]; M00962 (consensus sequence WGAGCANRN) is derived from 30 compiled AR regulated genes but only four of them are human genes [p21WAF1, PIGR (polymeric immunoglobulin receptor), AR and KLK3] (from the Transfac database). Non-consensus ARE sequences such as 5′-GTAAAGTACTCCAAGAA-3′ were previously identified for the androgen-regulated gene probasin [45], [46].

We therefore proceed to see whether we can identify novel AR consensus that explain the AR bindings in PC3-AR cells. We identified three novel AR matrices (Figure 4) that can be mapped to 1,448, 1,012, 317 AR binding regions respectively (Table S6–S7), which is much higher than the number of mapped AR binding regions using the three known canonical AR motifs from the Transfac database as we showed above. The consensus sequences of the new motif Gib1 and Gib2 are very similar, as both share a common core sequence motif CGAGCTCTTC. Motif Gib1 extends 3′ end two nucleotides (CG) and motif Gib2 extends 5′ end two nucleotides (TN) (N, any nucleotide) (Figure 4). The union of the AR binding regions containing one of these two motifs gives 1,808 AR binding regions, which explains 27.3% (1,808/6,629) of the AR binding events in PC3-AR cells. Our result suggests that the AR binding motif in PC3-AR cells might be very different from the canonical AR motif that was defined using androgen responsive prostate cells. Future mutagenesis and functional analysis is necessary to truly define these new AR motifs as functional AR binding motifs.

## Materials and Methods

### Chromatin IP

PC3 cells (catalogue # CRL-1435) were obtained from ATCC (http://www.atcc.org/). We transfected a full-length wild-type AR into PC3 prostate cancer cells that do not express the AR. The AR construct is a generous gift from Dr. Donald J. Tindall at the Mayo Clinic in Rochester. In this construct (pCMVhAR), the wide-type AR is placed under the CMV promoter. LipofectimineTM 2000 reagent (Promega Inc.) was used for transfection according to the manufacturer's protocol. For chromatin immunoprecipitation (ChIP), two anti-AR antibodies from BD Pharmagen were tested for their performance (data not shown). The specificities of the chromatin IP of the two AR antibodies were tested by PCR amplification of the promoter of the prostate specific antigen (PSA) gene, a gene that is directly regulated by the AR [47]. The antibody (BD Pharmagen Cat No. 554224) that has higher specificity was used (data not shown) in the final AR ChIP. For ChIP, 48 hours after transfection, the protein was cross-linked to DNA by adding formaldehyde according to the protocol by Upstate's chromatin immonoprecipitation kit. A no-antibody control chromatin IP was also performed. The DNAs isolated with anti-AR chromatin IP show a specific band corresponding to the PSA promoter, and the DNAs isolated with ‘no-antibody’ chromatin IP do not (Figure S1).

### ChIP-seq analysis

The chromatin IP DNA was digested with proteinase K and then purified by Qiagen Qiaquick PCR purification kit. ChIP DNA end repairing, adaptor ligation, and amplification were performed as described earlier [21]. Fragments of about 100 bp (without linkers) were isolated from agarose gel and used for sequencing using the Solexa/Illumina 1 G genetic analyzer.

### ChIP-seq data analysis

Solexa Pipeline Analysis was performed as described [21]. Sequence reads that map to multiple sites in the human genome were removed. The output of the Solexa Analysis Pipeline was converted to browser extensible data (BED) files for viewing the data in the UCSC genome browser. To identify AR binding regions, CisGenome was used for data analysis using two-sample mode with the AR ChIP-seq as positive and the IGG control ChIP-seq data as negative input. To calculate the distance to a TSS start site, annotations from the UCSC genome browser were used. We also took into the consideration of the direction of the strand when calculating the distance to TSS. As the AR binding regions is always recorded on the positive strands, for genes mapped to the positive strand, the distance is the end position of the AR binding region minus the TSS start position; for genes mapped to the negative strand, the distance is the TSS start position minus the AR binding region start position.

### Motif Scanning and identification

To scan for matched of single TF position-weight matrix, we used Cisgenome program using the “single matrix → FASTA” function. The matching criteria was set at likelihood ratio (LR)> = 500, and the order of background Markov Chain was set to three. We used the Gibbs Motif Sampler of the CisGenome program to identify novel AR binding motifs in the AR binding regions with a likelihood (LR) ratio of 500 compared to IGG ChIP-Seq background. For a systematic search for all potential transcription binding sites, we used the stand-alone motif scanner software (http://homes.esat.kuleuven.be/~thijs/download.html). Human upstream sequences from EPD (The Eukaryotic Promoter Database) (epd_homo_sapiens_499_chromgenes_non_split_3.bg) were downloaded from the motif scanner web site and used as the background model. The human subset of the Transfac professional 7.0 PWM matrices was used. Matched matrices with likelihood (LR) ratios of 500 or higher were tabulated and frequency calculated.

### Expression profiling and data analysis

PC3 cells were starved for 48 hours in androgen-deprived medium containing 10% dextran-filtered, charcoal-stripped fetal calf serum, and then transfected with pCMVhAR construct or vector alone (MOCK) using lipofectimineTM 2000 reagent (Promega Inc.) according to the manufacturer's protocol. Twenty-four hours after transfection, the culture media were replaced with culture media containing ETOH (solvent for R1881), or 1 nM R1881 or 10 nM R1881. Cells were harvested 48 hours transfection (i.e. 24 hours after change of media) and RNAs isolated according to the method we described previously [48]. We have performed two biological replicates for the MOCK condition, ETOH condition and the 1 nM condition. We compared the biological replicates and saw that they are highly consistent with correlation scores of 0.995, 0.993 and 0.995 respectively for all probes (Figure S2 and S3). For the 10 nM condition, we only completed one replicate due to lack of arrays from the same batch. As the biological replicates are highly reproducible, the data should be consistent and comparable to the other conditions.

Affymetrix U133Plus2 chips were used and standard array hybridization protocol as suggested by the manufacturer as used. Array data were normalized by the GCRMA method [49]. To determine present (absent) probesets, we applied a Gaussian mixture modeling method [50] to the normalized data set as follows: 1) Two Gaussian probability density functions (PDFs), one for ‘absent’ probesets and the other for ‘present’ probesets, were fitted to the distribution of the observed probeset intensities; and 2) Among 54,675 probesets, 26,056 probesets whose maximum intensities in all arrays were higher than the threshold where two fitted Gaussian PDFs meet were identified as present. The average normalized intensities were used for biological replicates (MOCK, ETOH and 1 nM conditions). For each experimental condition (ETOH, 1 nM and 10 nM conditions), present genes with absolute fold change higher than two were chosen as differentially expressed genes (DEGs)

### Statistical Analysis

We applied one-sided Fisher's exact test for testing the significant of enrichment. In additional, we also calculated the P values from Gaussian fitting using 10000 randomizations by randomly sampling. We identified 152 genes that overlap between the 2328 PC3 AR DEGs and 757 LNCAP AR DEGs. The union of these two gene lists gave a list of 2933 genes. The following procedure was performed to compute the probability that the number of overlapping genes in the two lists exceed the observed 152 genes by chance: 1) we randomly sampled 757 genes independently from 2933 AR-regulated genes, 2) the number of overlapping genes between the two randomly sampled gene sets was counted, 3) the above steps were repeated 10000 times, 4) an empirical probability density function was fitted to the 10000 numbers of overlapping genes from random experiments, 4) a p-value was computed as 1- normcdf (152,m,s) where m and s are the mean and standard deviation estimated from the normal distribution fitting.

## Supporting Information

Table S1 4166 differentially expressed genes in PC3-AR cells compared to Mock-transfected PC3 cells and compared to themselves at 1 nM and 10 nM androgen treatment conditions.
(0.85 MB XLS)

Table S2 Gene Ontology Enrichment analysis of differentially expressed genes.
(0.05 MB XLS)

Table S3 AR binding regions identified in PC3-AR cells.
(3.60 MB XLS)

Table S4 Known AR consensus matrice from the Transfac Database mapped to AR binding regions identified.
(0.01 MB TXT)

Table S5 Mapping of novel AR motif Gib1 to the AR binding regions of PC3-AR cells
(0.08 MB XLS)

Table S6 Mapping of novel AR motif Gib2 to the AR binding regions of PC3-AR cells.
(0.05 MB XLS)

Table S7 Mapping of novel AR motif Gib3 to the AR binding regions of PC3-AR cells.
(0.02 MB XLS)

Table S8 Ranking of known TF matches to the AR binding regions of PC3-AR cells identified by MotifScanner.
(0.06 MB XLS)

Table S9 Comparison of AR binding regions of PC3-AR cells and LNCaP cells within 1.5 kb of TSS sites.
(0.05 MB XLS)

Figure S1 ChIP with the AR antibody generated a band that is the same size as the positive (input) control. ChIP without the primary AR antibody (without Ab) but with the 2nd antibody IgG alone generate no-specific PCR product, suggesting the AR ChIP is specific. The negative control (no template) showed negative. The bottom bands across the lanes are primer dimmer.
(0.15 MB TIF)

Figure S2 Quality control scatter plot of replicate array hybridization showing the replicates are of good qualities.
(1.68 MB TIF)

Figure S3 Scatter plot comparing Mock (empty vector) vs. AR transfected PC3 cells in different androgen conditions. When compared with the scatter plot of the replicates, differential expression of genes is evident.
(1.72 MB TIF)
