# Supplementary figures and images for: Integrated Expression Profiling and ChIP-seq Analyses of the Growth Inhibition Response Program of the Androgen Receptor
Source: PLoS One. 2009 Aug 11;4(8):e6589. doi: 10.1371/journal.pone.0006589 (PMC2720376; doi:10.1371/journal.pone.0006589)

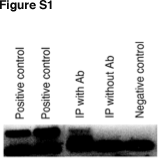

Supplement: Figure S1 — ChIP with the AR antibody generated a band that is the same size as the positive (input) control. ChIP without the primary AR antibody (without Ab) but with the 2nd antibody IgG alone generate no-specific PCR product, suggesting the AR ChIP is specific. The negative control (no template) showed negative. The bottom bands across the lanes are primer dimmer. (0.15 MB TIF) [file pone.0006589.s010.tif]

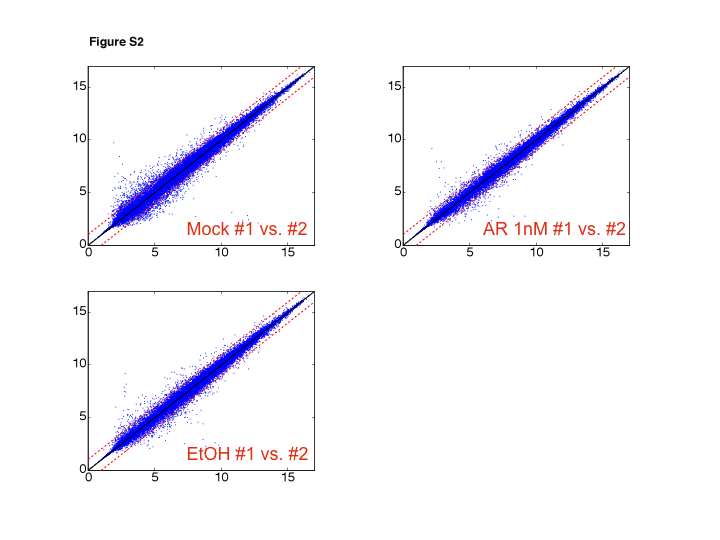

Supplement: Figure S2 — Quality control scatter plot of replicate array hybridization showing the replicates are of good qualities. (1.68 MB TIF) [file pone.0006589.s011.tif]

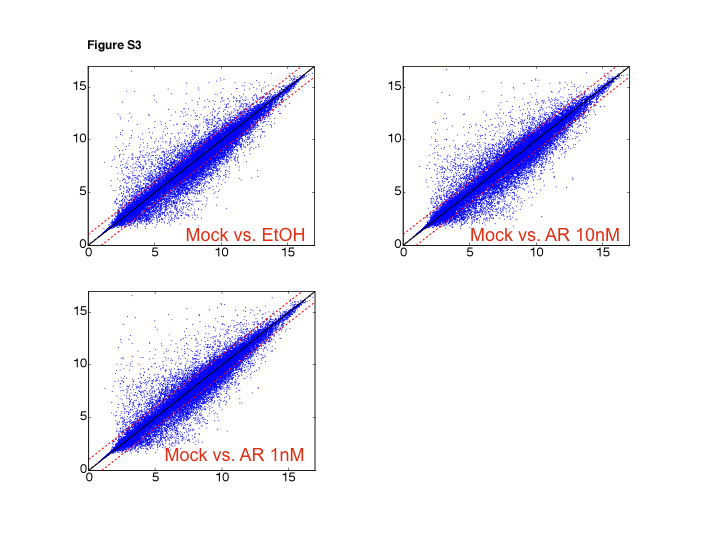

Supplement: Figure S3 — Scatter plot comparing Mock (empty vector) vs. AR transfected PC3 cells in different androgen conditions. When compared with the scatter plot of the replicates, differential expression of genes is evident. (1.72 MB TIF) [file pone.0006589.s012.tif]
